# Association between obstructive sleep apnea and arrhythmia and heart rate variability among hypertensive patients

**DOI:** 10.1186/s12872-024-04008-5

**Published:** 2024-07-04

**Authors:** Shao-dong Xu, Ling-li Hao, Fei-fei Liu, Chuan-zhi Xu

**Affiliations:** 1 Department of Cardiology, The Third Affiliated Hospital of Anhui Medical University, Hefei, Anhui Province 230001 China; 2 Department of Sleep Monitoring Center, The Third Affiliated Hospital of Anhui Medical University, Hefei, Anhui Province 230001 China; 3 Department of Electrocardiogram, The Third Affiliated Hospital of Anhui Medical University, Hefei, Anhui Province 230001 China

**Keywords:** Obstructive sleep apnea (OSA), Arrhythmia, Heart rate variability (HRV), Hypertension

## Abstract

**Background:**

The relationship between obstructive sleep apnea (OSA) and the occurrence of arrhythmias and heart rate variability (HRV) in hypertensive patients is not elucidated. Our study investigates the association between OSA, arrhythmias, and HRV in hypertensive patients.

**Methods:**

We conducted a cross-sectional analysis involving hypertensive patients divided based on their apnea-hypopnea index (AHI) into two groups: the AHI ≤ 15 and the AHI > 15. All participants underwent polysomnography (PSG), 24-hour dynamic electrocardiography (DCG), cardiac Doppler ultrasound, and other relevant evaluations.

**Results:**

The AHI > 15 group showed a significantly higher prevalence of frequent atrial premature beats and atrial tachycardia (*P* = 0.030 and *P* = 0.035, respectively) than the AHI ≤ 15 group. Time-domain analysis indicated that the standard deviation of normal-to-normal R-R intervals (SDNN) and the standard deviation of every 5-minute normal-to-normal R-R intervals (SDANN) were significantly higher in the AHI > 15 group (*P* = 0.020 and *P* = 0.033, respectively). Frequency domain analysis revealed that the low-frequency (LF), high-frequency (HF) components, and the LF/HF ratio were also significantly elevated in the AHI > 15 group (*P* < 0.001, *P* = 0.031, and *P* = 0.028, respectively). Furthermore, left atrial diameter (LAD) was significantly larger in the AHI > 15 group (*P* < 0.001). Both univariate and multivariable linear regression analyses confirmed a significant association between PSG-derived independent variables and the dependent HRV parameters SDNN, LF, and LF/HF ratio (*F* = 8.929, *P* < 0.001; *F* = 14.832, *P* < 0.001; *F* = 5.917, *P* = 0.016, respectively).

**Conclusions:**

Hypertensive patients with AHI > 15 are at an increased risk for atrial arrhythmias and left atrial dilation, with HRV significantly correlating with OSA severity.

## Background

Hypertension, a prevalent cardiovascular condition, induces cardiac hypertrophy, atrial enlargement, and over-excitation of the renin-angiotensin-aldosterone system (RAAS) and sympathetic nervous system [1–5]. These physiological changes can precipitate a range of arrhythmias. Concurrently, obstructive sleep apnea (OSA), characterized by intermittent nocturnal apneas and hypopneas, leads to recurrent episodes of hypoxemia, hypercapnia, and sleep disruption [6, 7]. Such disturbances have been implicated in the genesis of diverse arrhythmias and alterations in heart rate variability (HRV) [8, 9]. The coexistence of hypertension and OSA is not uncommon and poses a complex interplay between the two conditions [10–12].

Our previous findings have highlighted that OSA can exacerbate gut microbiota imbalance in hypertensive patients, triggering inflammatory responses [13]. Furthermore, hypertensive individuals with moderate to severe OSA have demonstrated a marked increase in blood pressure variability (BPV) and blood pressure load (BPL) [14]. While evidence suggests that OSA may intensify heart rhythm and rate abnormalities [15–17], the precise relationship between OSA and the occurrence of arrhythmias and HRV in hypertensive patients remains to be elucidated.

Heart rate regulation is a complex process influenced by neural and humoral factors. HRV is a reliable indicator of autonomic nervous system function, and its assessment is particularly pertinent in the context of OSA [18, 19]. The ambulatory electrocardiogram (DCG) is a widely utilized clinical tool for evaluating arrhythmias and heart rate dynamics.

The present study aims to explore the relationship between OSA and the occurrence of arrhythmias and HRV in hypertensive patients, which seeks to deepen our understanding of the interaction between cardiac and nervous system dysfunction in hypertensive patients with OSA. Our study can potentially refine clinical practice, ultimately enhancing diagnostic accuracy and treatment strategies for hypertensive patients with OSA.

## Methods

### Study population

Our study was a cross-sectional study that enrolled a total of 265 hypertensive patients who were randomly hospitalized between February 2021 and May 2022. The exclusion criteria included patients with bronchial asthma, chronic obstructive pulmonary disease, acute illnesses, and those who consumed coffee or used sedative drugs. Additionally, patients with central or mixed OSA, secondary hypertension unrelated to OSA, acute myocardial infarction, acute cerebral infarction or cerebral hemorrhage, and tumors were also excluded. All enrolled patients received standardized and individualized antihypertensive treatments.

### Baseline clinical characteristics collection

Baseline data collection encompassed the recording of demographic variables, including Age and Gender, along with clinical parameters such as Current smoking status, Coronary heart disease, Cerebral infarction, Chronic heart failure, and Chronic kidney disease. Additionally, anthropometric measurements were obtained, including Body Mass Index (BMI), and relevant laboratory assessments comprised glomerular filtration rate (GFR), blood glucose, creatinine, total triglycerides (TG), high-density lipoprotein cholesterol (HDL-C), low-density lipoprotein cholesterol (LDL-C), and total cholesterol (TC). The study also documented the utilization of various antihypertensive medications.

### Polysomnography (PSG) monitoring

During hospitalization, an 8-hour polysomnography (PSG) monitoring session was conducted and reported by certified professionals from the hospital sleep center. PSG involves recording nasal and oral airflow, snoring, oxygen saturation levels, electrocardiogram (ECG) readings, thoracic and abdominal movements, body movements, and finger pulse data. Key parameters such as minimum oxygen saturation, total occurrences of oxygen reduction, instances of apnea, total apnea duration, apnea index, average hypoventilation duration, and hypoventilation index were meticulously recorded and subsequently analyzed by trained professionals. These professionals adhere to strict standardization protocols to ensure the reliability and validity of the data collected. The AHI, representing the total number of sleep apnea and hypopnea events per hour, was calculated but remained undisclosed until the conclusion. Based on the AHI values, participants were stratified into two groups: those with AHI ≤ 15 and those with AHI > 15.

### 24-hour DCG monitoring

During hospitalization, all participants underwent DCG monitoring using the DMS300-4 device by certified professionals from the hospital electrocardiogram room, adhering to standardized reporting guidelines to maintain high standards. The monitoring system captured and analyzed various parameters related to cardiac rhythm, including the shape, duration, and frequency of different arrhythmias. The detected data were automatically stored, processed, and corrected by computer algorithms, after which the results were generated in print format. Tachyarrhythmias encompass conditions such as premature atrial beats, premature ventricular beats, borderline premature beats, atrial tachycardia, ventricular tachycardia, and cross-bound tachycardia. On the other hand, bradyarrhythmias include sinus arrest beats, sinus blocks, atrioventricular blocks, and similar conditions.

### Heart rate variability (HRV)

During the DCG monitoring, the time and frequency domain parameters of HRV were analyzed. The time domain parameters included the standard deviation of normal-to-normal R-R intervals (SDNN) and the standard deviation of every 5-minute normal-to-normal R-R intervals (SDANN). The frequency domain parameters included high frequency (HF), low frequency (LF), and the LF/HF ratio. SDNN and SDANN reflect overall autonomic variability, with SDNN indicating short-term variability and SDANN representing long-term variability. LF is associated with both sympathetic and parasympathetic activity, while HF is primarily related to parasympathetic activity. The LF/HF ratio indicates the balance between sympathetic and parasympathetic influences on heart rate.

### Echocardiogram

All participants underwent cardiac Doppler ultrasound examination using a probe frequency ranging from 2.5 to 4.0 MHz as part of the experimental procedures. The examination specifically included the measurement of left atrial diameter, a key parameter used to assess cardiac structure and function.

### Statistical analysis

Data processing was conducted using IBM SPSS Statistics 26.0, while graphical representations were created with GraphPad Prism 8. Outliers were identified using residual examination and were carefully evaluated and excluded to ensure the robustness of the analysis. Statistical tests were chosen based on the data characteristics and research questions. Continuous variables, confirmed to meet assumptions of normality and homogeneity of variances, were analyzed using Student’s *t*-tests, and results were expressed as mean ± standard deviation (SD). Categorical variables were evaluated for baseline comparability via the chi-square test or Fisher’s exact test, with results presented as percentages (%). Univariate linear regression was utilized to investigate the relationships between independent variables derived from PSG characteristics and the dependent variables SDNN, LF, and LF/HF ratio. Significant variables from the univariate analysis were subsequently included in a multiple-stepwise linear regression model to determine their predictive power, with the total sample size being at least ten times the number of independent variables. A significance threshold of *P* < 0.05 was maintained for all statistical tests.

## Results

### Baseline clinical characteristics of the participants

As depicted in Table 1, a comparative analysis of the baseline clinical characteristics between the AHI ≤ 15 and AHI > 15 groups revealed no significant differences across most parameters. Nonetheless, notable disparities were observed in Age, Gender, BMI, and the utilization of Antihypertensive medications. The AHI > 15 group exhibited a younger age and a higher proportion of males (*P* = 0.043 and 0.005, respectively) than the AHI ≤ 15 group. Moreover, the AHI > 15 group showed a more pronounced increase in BMI (*P* < 0.001) and greater utilization of various antihypertensive medications, including β-blockers and angiotensin receptor neprilysin inhibitors (*P* = 0.015 and 0.039, respectively), compared to the AHI ≤ 15 group.

**Table 1:** Comparison of baseline clinical characteristics of the participants

Characteristics	AHI ≤ 15 (*n* = 114)	AHI > 15 (*n* = 151)	t/χ²	*P* value
Age	61.70 ± 12.61	58.43 ± 13.17	2.038	0.043
Male (%)	66 (57.89)	112 (74.17)	7.805	0.005
BMI	25.48 ± 2.97	28.19 ± 4.44	-5.941	<0.001
Blood glucose	5.49 ± 1.70	5.85 ± 1.83	-1.623	0.106
Creatinine	74.52 ± 19.75	76.68 ± 17.54	-0.936	0.350
Cystatin	0.83 ± 0.32	0.86 ± 0.35	-0.878	0.381
Glomerular filtration rate	92.78 ± 17.04	94.87 ± 17.38	-0.973	0.331
TG (mmol/L)	2.02 ± 2.25	2.38 ± 2.37	-1.271	0.205
TC (mmol/L)	4.14 ± 1.11	4.35 ± 1.10	-1.527	0.128
LDL-C (mmol/L)	2.17 ± 0.97	2.31 ± 1.09	-1.066	0.287
HDL-C (mmol/L)	1.16 ± 0.32	1.08 ± 0.27	2.068	0.040
Current smoking (%)	26 (22.81)	40 (26.49)	0.471	0.492
Medical history				
Diabetes (%)	25 (21.93)	30 (19.87)	0.168	0.682
Coronary heart disease (%)	23 (20.18)	32 (21.19)	0.041	0.840
Cerebral infarction (%)	69 (60.53)	82 (54.30)	1.026	0.311
Chronic heart failure (%)	2 (1.75)	8 (5.30)	2.246	0.196
Chronic kidney disease (%)	1 (0.88)	5 (3.31)	1.739	0.241
Antihypertensive drugs				
Calcium channel blockers (%)	92 (80.70)	134 (88.74)	3.346	0.067
Angiotensin-converting enzyme inhibitor (%)	11 (7.80)	10 (6.62)	0.816	0.366
Angiotensin II receptor blockers (%)	57 (50)	68 (45.03)	0.643	0.423
β-Blockers (%)	29 (25.44)	60 (39.74)	5.952	0.015
Angiotensin receptor neprilysin inhibitor (%)	8 (7.02)	23 (15.23)	4.243	0.039
Diuretic (%)	12 (15.23)	19 (12.58)	0.266	0.606
Others (%)	2 (1.75)	3 (1.99)	0.019	1.000

Note: Categorical characteristics were presented as number (percentage), and continuous characteristics were presented as mean ± standard deviation (SD). BMI: body mass index; TG: total glyceride; TC: total cholesterol; LDL-C: low density cholesterol; HDL-C: high density cholesterol

### PSG characteristics of the participants

Table 2 highlights the statistical differences between the AHI ≤ 15 and AHI > 15 groups across various parameters, such as the Mean time of apnea, Times of hypoventilation, and Hypoventilation index (*P* < 0.05). Notably, the two groups have no significant difference in the Mean time of hypoventilation.

**Table 2:** Comparison of PSG characteristics in two groups

Characteristics	AHI ≤ 15 (*n* = 114)	AHI>15 (*n* = 151)	t	*P* value
Minimum oxygen saturation	85.96 ± 6.32	76.23 ± 10.79	9.200	<0.001
Mean oxygen saturation	95.37 ± 1.44	93.49 ± 3.07	6.637	<0.001
Total times of oxygen reduction	70.15 ± 40.36	272.59 ± 132.34	-17.737	<0.001
Oxygen reduction index	8.77 ± 5.01	38.30 ± 31.90	-11.194	<0.001
Times of apnea	23.58 ± 39.30	189.28 ± 142.82	-13.592	<0.001
Mean time of apnea	17.05 ± 9.28	24.96 ± 7.44	-7.697	<0.001
Total apnea time	8.60 ± 14.95	86.18 ± 77.09	-12.069	<0.001
Maximum apnea time	29.92 ± 21.43	53.62 ± 22.99	-8.551	<0.001
Apnea index	2.83 ± 5.07	24.29 ± 18.49	-13.602	<0.001
Times of hypoventilation	41.45 ± 32.40	94.88 ± 64.32	-8.831	<0.001
Mean time of hypoventilation	23.89 ± 6.09	24.60 ± 7.84	-0803	0.423
Total hypoventilation time	17.24 ± 14.85	37.96 ± 24.49	-8.528	<0.001
Maximum hypoventilation time	44.20 ± 16.62	51.43 ± 21.69	-2.962	0.003
Hypoventilation index	5.14 ± 4.18	12.49 ± 8.52	-9.232	<0.001

Note: Continuous characteristics were presented as mean ± standard deviation (SD)

### Arrhythmia and HRV of the participants

Table 3 demonstrates that compared to the AHI ≤ 15 group, the AHI > 15 group exhibited higher rates of Frequent atrial premature beat and Atrial tachycardia (*P* = 0.030 and 0.035, respectively).

**Table 3:** Comparison of arrhythmia in two groups

Characteristics	AHI ≤ 15 (*n* = 114)	AHI>15 (*n* = 151)	χ²	*P* value
Frequent atrial premature beat (%)	11 (9.65)	29 (19.21)	4.630	0.030
Atrial tachycardia (%)	27 (23.68)	54 (35.76)	4.464	0.035
Atrial fibrillation (%)	4 (3.51)	6 (3.97)	0.035	0.852
Frequent ventricular premature beat (%)	8 (7.02)	11 (7.28)	0.007	0.933
Sinus arrest (%)	0 (0)	1 (0.66)	-	-
Atrioventricular block (%)	2 (1.75)	6 (3.97)	1.093	0.472

Note: Categorical characteristics were presented as number (percentage)

Table 4 demonstrates that compared with the AHI ≤ 15 group, the AHI > 15 group exhibited higher values for certain time domain parameters, SDNN and SDANN (*P* = 0.020 and 0.033, respectively), as well as certain frequency domain parameters, LF, HF, and LF/HF ratio (*P* < 0.001, 0.031, and 0.028, respectively).

**Table 4:** Comparison of HRV in two groups

Characteristics	AHI ≤ 15 (*n* = 114)	AHI>15 (*n* = 151)	t	*P* value
SDNN	120.96 ± 29.45	130.89 ± 39.91	2.332	0.020
SDANN	102.94 ± 25.92	111.3 ± 37.65	2.139	0.033
LF	314.15 ± 172.33	442.37 ± 257.05	4.853	< 0.001
HF	211.06 ± 136.67	254.68 ± 190.96	2.167	0.031
LF/HF	1.92 ± 1.20	2.66 ± 2.96	2.770	0.006

Note: Continuous characteristics were presented as mean ± standard deviation (SD). HRV, Heart rate variability; SDNN, the standard deviation of normal-to-normal R-R intervals; SDANN: the standard deviation of every 5-minute normal-to-normal R-R intervals; LF: low frequency; HF: high frequency

### Regression analysis of HRV parameters and PSG characteristics

As summarized in Tables 5, 6 and 7, the univariate linear regression analyses evaluated the relationships between PSG characteristics and the dependent variables SDNN, LF, and LF/HF ratio. Variables that reached a significance level of *P* < 0.05 were further analyzed using multiple stepwise linear regression models. The ANOVA for these models confirmed statistical significance, with *F*-values of 8.929 (*P* < 0.001), 14.832 (*P* < 0.001), and 5.917 (*P* = 0.016), respectively, indicating a significant linear relationship between the dependent and independent variables. The multivariable linear regression model for SDNN demonstrated an adjusted *R*-squared of 0.029 (*R* = 0.181, *R*-squared = 0.033), suggesting minimal but significant predictive power. The model identified ‘total times of oxygen reduction’ as a significant predictor (Table 5, regression coefficient = 0.045, *P* = 0.003). For the LF parameter, the adjusted *R*-squared was higher at 0.050 (*R* = 0.231, *R*-squared = 0.053). The ‘total times of oxygen reduction’ again emerged as a significant contributor to the model (Table 6, regression coefficient = 0.374, *P* < 0.001). The adjusted *R*-squared for the LF/HF ratio model was 0.024 (*R* = 0.165, *R*-squared = 0.027), with ‘times of apnea’ identified as a significant factor (Table 7, regression coefficient = 0.020, *P* = 0.007). These findings highlight the impact of specific PSG characteristics on HRV parameters in individuals with OSA, demonstrating the nuanced relationships between sleep disruptions and autonomic cardiac control.

**Table 5:** Univariate and multivariable linear regression analysis of candidate risk factors associated with SDNN

Characteristics	Univariate linear regression analysis				Multivariable linear regression analysis			
	Estimate	SE	t	*P*	Estimate	SE	t	*P*
Apnea index	0.307	0.123	2.497	0.013				
Hypoventilation index	0.186	0.282	0.659	0.510				
Minimum oxygen saturation	-0.387	0.214	-1.806	0.072				
Mean oxygen saturation	-1.842	0.826	-2.230	0.027				
Total times of oxygen reduction	0.045	0.015	2.988	0.003	0.045	0.015	2.988	0.003
Oxygen reduction index	0.090	0.078	1.145	0.253				
Times of apnea	0.041	0.016	2.558	0.011				
Mean time of apnea	0.566	0.241	2.354	0.019				
Total apnea time	0.081	0.031	2.590	0.010				
Maximum apnea time	0.239	0.087	2.741	0.007				
Times of hypoventilation	0.031	0.038	0.826	0.410				
Mean time of hypoventilation	-0.094	0.312	-0.300	0.764				
Total hypoventilation time	0.063	0.096	0.662	0.509				
Maximum hypoventilation time	0.056	0.111	0.498	0.619				
AHI	0.278	0.106	2.617	0.009				

Note: AHI: apnea hypopnea index

**Table 6:** Univariate and multivariable linear regression analysis of candidate risk factors associated with LF

Characteristics	Univariate linear regression analysis				Multivariable linear regression analysis			
	Estimate	SE	t	*P*	Estimate	SE	t	*P*
Apnea index	2.628	0.788	3.332	0.001				
Hypoventilation index	3.475	1.812	1.918	0.056				
Minimum oxygen saturation	-3.015	1.379	-2.185	0.030				
Mean oxygen saturation	-4.289	5.381	-0.797	0.426				
Total times of oxygen reduction	0.374	0.097	3.851	< 0.001	0.374	0.097	3.851	< 0.001
Oxygen reduction index	1.038	0.503	2.065	0.040				
Times of apnea	0.343	0.102	3.363	0.001				
Mean time of apnea	2.767	1.561	1.772	0.077				
Total apnea time	0.630	0.200	3.145	0.002				
Maximum apnea time	1.018	0.567	1.797	0.074				
Times of hypoventilation	0.519	0.241	2.156	0.032				
Mean time of hypoventilatin	-2.194	2.009	-1.092	0.276				
Total hypoventilation time	0.745	0.616	1.209	0.228				
Maximum hypoventilation time	0.616	0.719	0.857	0.392				
AHI	2.509	0.678	3.700	< 0.001				

Note: AHI: apnea hypopnea index

**Table 7:** Univariate and multivariable linear regression analysis of candidate risk factors associated with LF/HF

Characteristics	Univariate linear regression analysis				Multivariable linear regression analysis			
	Estimate	SE	t	*P*	Estimate	SE	t	*P*
Apnea index	0.013	0.006	2.327	0.021				
Hypoventilation index	0.019	0.013	1.554	0.121				
Minimum oxygen saturation	-0.009	0.010	-0.933	0.351				
Mean oxygen saturation	-0.041	0.037	-1.100	0.272				
Total times of oxygen reduction	0.001	0.001	1.368	0.173				
Oxygen reduction index	0.004	0.003	1.148	0.252				
Times of apnea	0.001	0.001	1.995	0.047				
Mean time of apnea	0.016	0.011	1.507	0.133				
Total apnea time	0.003	0.001	2.016	0.045				
Maximum apnea time	0.006	0.004	1.436	0.152				
Times of hypoventilation	0.003	0.002	1.707	0.089				
Mean time of hypoventilatin	0.021	0.014	1.538	0.125				
Total hypoventilation time	0.008	0.004	1.880	0.061				
Maximum hypoventilation time	0.010	0.005	2.103	0.036				
AHI	0.012	0.005	2.432	0.016	0.012	0.005	2.432	0.016

Note: AHI: apnea hypopnea index

### Comparison of left atrial diameter in two groups

**Fig. 1:**
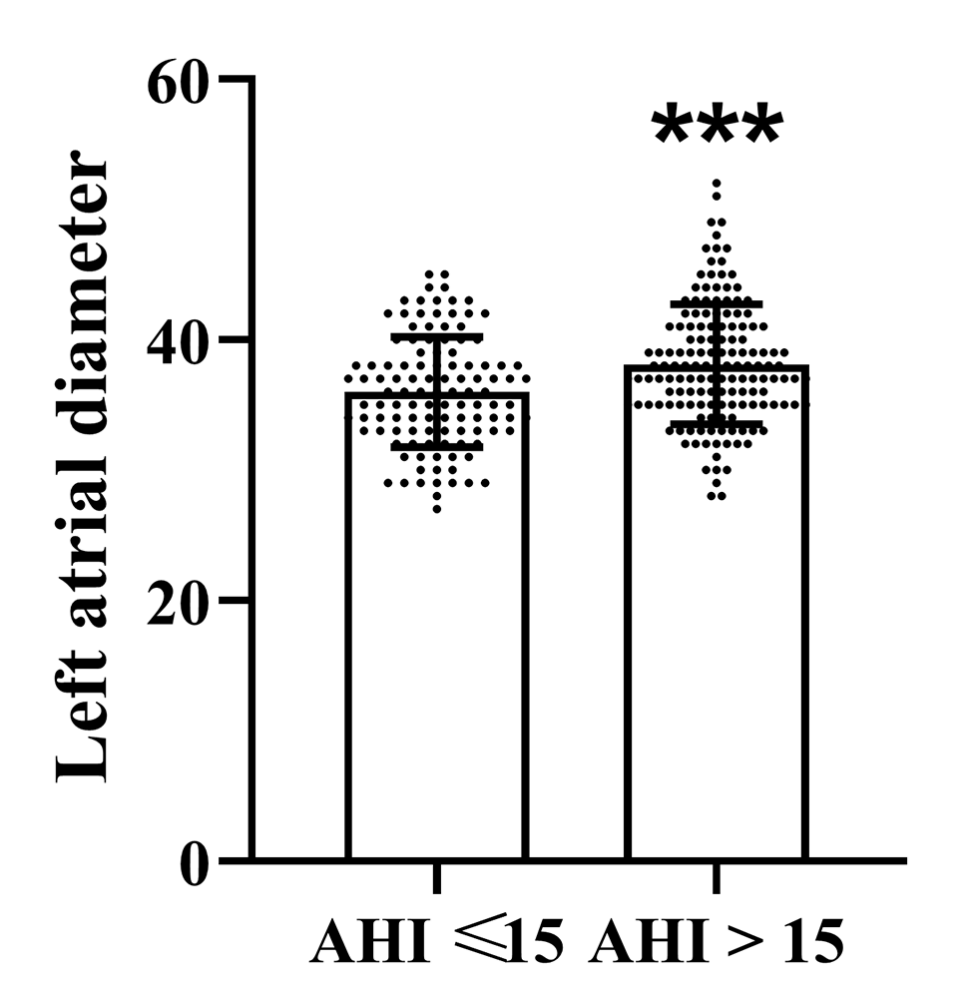
Comparison of left atrial diameter between patients with AHI ≤ 15 and AHI > 15. The figure shows a significant increase in left atrial diameter in the AHI > 15 group compared to the AHI ≤ 15 group, based on echocardiographic measurements. The data indicate a strong association between higher AHI and left atrial dilation. Statistical analysis confirmed the significance of these findings, with ^***^P < 0.001

Figure 1 shows a significant increase in left atrial diameter in the AHI > 15 group compared to the AHI ≤ 15 group (*P* < 0.001), which indicates a strong association between higher AHI and left atrial dilation.

## Discussion

In the present study, individuals with AHI > 15 displayed a significantly increased prevalence of atrial premature beats and atrial tachycardia. Notably, both time-domain measures (SDNN and SDANN) and frequency-domain components (LF, HF, and the LF/HF ratio) were markedly elevated in the AHI > 15 group. Additionally, substantial enlargement of the LAD was observed among these individuals. Linear regression analysis confirmed a strong association between PSG characteristics and the HRV parameters SDNN, LF, and LF/HF ratio. Our findings suggest that AHI > 15 contributes significantly to cardiac electrophysiological and structural characteristics alterations.

The prevalence of OSA varies among hypertensive patients, with a significant proportion of middle-aged individuals affected [20]. Consistent with the previous study [21], we observed a higher prevalence of OSA in males and an association with increased BMI in the hypertensive population with AHI > 15. Notably, the younger age and higher male predominance in the AHI > 15 group contradict the common perception that advancing age directly correlates with OSA severity. The increased use of antihypertensive medications, including β-blockers and angiotensin receptor neprilysin inhibitors, in the AHI > 15 group may suggest more challenging blood pressure management in these patients.

Our findings align with prior research indicating that hypertensive patients with moderate to severe OSA exhibit increased BPV and BPL [14]. It is well-documented that chronic hypertension is known to precipitate various arrhythmias, and OSA has been closely linked to their occurrence [20]. A seminal study by Almeneessier et al., which evaluated 394 individuals with OSA, revealed a heightened incidence of arrhythmias, including premature atrial contractions (PACs) and premature ventricular contractions (PVCs), within OSA patients [9]. In alignment with these findings, our study observed an increased frequency of atrial premature beats and atrial tachycardia. However, contrary to expectations, the frequent ventricular premature beat did not register a significant rise in hypertensive patients with AHI > 15.

Several mechanisms may underpin these observations. The recurrent episodes of apnea and hypopnea characteristic of OSA, particularly in patients with an AHI > 15, result in severe hypoxemia, hypercapnia, reoxygenation, autonomic nervous system dysfunction, arousal, and sleep deprivation. These factors can collectively impact cardiac function and electrical stability [22]. Furthermore, the sympathetic overactivity, heightened reactivity of the RAAS, and myocardial hypertrophy, which are consequences of long-standing hypertension, exacerbate the damage to the heart’s target organs. Such conditions can readily facilitate left atrial remodeling [23, 24], ultimately precipitating the onset of atrial arrhythmias [25]. Our findings also corroborate an enlargement of the left atrial diameter in hypertensive patients with an AHI > 15.

Bazan et al. examined 77 patients experiencing newly onset atrial fibrillation within a month and identified OSA as a significant risk factor [26]. Similarly, Anter et al. found that 43 patients with OSA (AHI > 15) and paroxysmal atrial fibrillation exhibited lower atrial voltage amplitudes, slower conduction velocities, and a higher prevalence of electrogram fractionation [27]. In contrast, our study observed no significant differences in atrial fibrillation incidence, potentially because some patients were hospitalized for atrial fibrillation symptoms rather than hypertension. Consequently, these patients who received specific interventions like radiofrequency ablation during hospitalization were not included in our study, possibly reducing the observed incidence of atrial fibrillation.

Based on previous research and our findings, we believe that hypertension combined with OSA, especially in patients with an AHI > 15, significantly impacts arrhythmias and heart rate variability. It is essential to address OSA in addition to treating arrhythmias and hypertension. Implementing methods to reduce OSA severity, such as continuous positive airway pressure (CPAP) therapy, can improve arrhythmias and heart rate variability, thereby enhancing the overall prognosis for these patients.

HRV reflects minute variations between consecutive heartbeats, primarily governed by autonomic modulation via the sympathetic and parasympathetic nerves, with beat-to-beat differences generally spanning tens of milliseconds [28]. HRV analysis provides indirect quantitative insights into myocardial autonomic balance and can assess autonomic nervous system activity [29]. Previous research indicates that HRV metrics can effectively represent autonomic function in patients with OSA [18, 19]. HRV encompasses various parameters, both in time and frequency domains. For instance, SDNN and LF/HF ratios depict overall autonomic tone and balance; HF represents parasympathetic activity, while SDANN and LF indicate sympathetic tone [28]. The physiological basis for the observed changes in HRV can be attributed to the chronic intermittent hypoxia and sleep fragmentation characteristic of OSA, leading to increased sympathetic activity and reduced parasympathetic activity. Hypertension further exacerbates this imbalance, potentially leading to adverse cardiovascular outcomes such as arrhythmias. By examining these HRV parameters, our study aims to elucidate the autonomic dysfunction in hypertensive patients with OSA, thereby providing insights that could inform more effective management strategies.

In our study, we observed significant increases in SDNN among hypertensive patients with AHI > 15, suggesting enhanced overall autonomic activity. Specifically, increases in SDANN and LF indicate heightened sympathetic nerve activity, while elevated HF values suggest increased parasympathetic nerve activity. Consequently, these findings imply that both sympathetic and parasympathetic nervous systems are overstimulated in hypertensive patients with significant OSA, contributing to notable alterations in HRV. Univariate and multivariate linear regression analyses revealed significant associations between HRV indices (SDNN, LF/HF ratio) and polysomnographic features, including total hypoxic events and AHI. These findings underscore a robust correlation between HRV alterations and both the frequency and severity of OSA in hypertensive patients with AHI > 15.

The proposed mechanisms underlying these observations involve a cascade of physiological responses initiated by airway obstruction and increased respiratory resistance, leading to prolonged episodes of apnea and hypopnea [30]. These episodes induce sustained hypoxemia and hypercapnia, which in turn trigger sympathetic hyperactivity, particularly noticeable at the termination of apneic episodes [31, 32]. Following these periods, the body experiences acute hypoxia, which overstimulates the sympathetic nervous system. The relief of airway obstruction through ventilatory drive activation leads to a rapid decrease in respiratory resistance and transient hyperventilation, quickly correcting the hypoxemia.

Simultaneously, the induced shortness of breath results in respiratory alkalosis, stimulating parasympathetic nerve activation. However, this response also inhibits respiratory muscles, exacerbating the airway obstruction [33]. As cycles of apnea and hypopnea resume, the body quickly shifts from elevated parasympathetic to sympathetic activity. This recurring pattern of nerve excitation throughout the night causes significant fluctuations in autonomic nerve activity, ultimately reflected as increased HRV. These dynamics highlight the intricate relationships between respiratory disruptions and autonomic regulation in hypertensive patients with severe OSA (AHI > 15) [34–36]. In addition, regular monitoring of circadian blood pressure variations could improve patient outcomes by addressing the increased risk of diabetes associated with non-dipping patterns [12].

Our study highlights the need for enhanced clinical management of hypertensive patients with OSA, particularly those with an AHI > 15. Regular screening for OSA in hypertensive patients should be implemented, especially for those not responding well to conventional treatments. Integrating CPAP therapy and lifestyle modifications into treatment plans can improve both OSA severity and cardiovascular outcomes. A multidisciplinary approach involving cardiologists, pulmonologists, and sleep specialists is recommended to ensure comprehensive care. Regular monitoring of heart rate variability and arrhythmias can aid in timely treatment adjustments, ultimately improving the prognosis and quality of life for these patients.

In our study, the cross-sectional design limits our ability to establish temporal relationships and infer causality. Although we observed associations between hypertension, OSA severity, arrhythmias, and HRV, we cannot determine the direction of these relationships. There is a possibility of reverse causality; for instance, pre-existing arrhythmias might exacerbate OSA or hypertension rather than the other way around. Future longitudinal studies are necessary to clarify these temporal relationships and better understand the causal pathways involved. Such research would provide more definitive evidence on how OSA severity impacts cardiovascular health in hypertensive patients.

The present study, however, has several limitations. The cross-sectional design limits our ability to infer causality, as we can only establish associations, not cause-and-effect relationships. The small sample size and the fact that it was conducted at a single center may limit the generalizability of the findings. Future studies with larger, multi-center cohorts are necessary to validate and expand upon our results, ensuring they are applicable to a broader population. Additionally, only a single 24-hour DCG exam was performed for each patient, which might not fully capture the comprehensive cardiac status of the individuals involved. To enhance the validity of future research, increasing the monitoring duration and frequency of ambulatory ECG is recommended to better represent the real-time dynamics of arrhythmias and HRV in this patient population. Lastly, it is important to note that the study participants were all hospitalized due to exacerbations of their conditions, which may not accurately represent the broader population of hypertensive patients.

## Conclusions

Our study demonstrates that hypertensive patients with AHI > 15 exhibits significantly higher incidences of atrial arrhythmias and altered HRV parameters compared to those with an AHI ≤ 15. Both single and multiple regression analyses confirm that the degree of HRV alteration strongly correlates with the severity of OSA. These findings indicate that in hypertensive patients with substantial OSA, there is a tendency for atrial arrhythmias, as well as overexcitation of sympathetic and parasympathetic nerves, and left atrial enlargement. Thus, the changes in HRV are closely linked to the severity of OSA, underscoring the potential clinical importance of HRV monitoring in this patient population. Our study highlights the underexplored link between OSA severity, arrhythmias, and heart rate variability, offering new insights into cardiovascular impacts in OSA patients.

## Data Availability

The datasets used and/or analysed during the current study are available from the corresponding author on reasonable request.

## Abbreviations

OSA: obstructive sleep apnea;
HRV: heart rate variability;
AHI: apnea-hypopnea index;
PSG: polysomnography;
DCG: dynamic electrocardiogram;
SDNN: standard deviation of normal-to-normal R-R intervals;
SDANN: standard deviation of every 5-minute normal-to-normal R-R intervals;
LF: low frequency;
HF: high frequency;
LAD: left atrial diameter;
RAAS: renin angiotensin aldosterone system;
BPV: blood pressure variability;
BPL: blood pressure load;
BMI: body mass index;
GFR: glomerular filtration rate;
TG: total glyceride;
HDL-C: high-density cholesterol;
LDL-C: low-density cholesterol;
TC: total cholesterol;
ECG: electrocardiogram;
SD: standard deviation;
PACs: premature atrial contractions;
PVCs: premature ventricular contractions;
CPAP: continuous positive airway pressure
